# Comprehensive study of the mountainous lake sediments in relation to natural and anthropogenic processes and time (Mały Staw Lake, Poland)

**DOI:** 10.1007/s11356-017-0711-x

**Published:** 2017-11-17

**Authors:** Katarzyna Szarlowicz, Witold Reczynski, Agnieszka Czajka, Barbara Spyt, Grzegorz Szacilowski

**Affiliations:** 1 AGH University of Science and Technology, Faculty of Energy and Fuels, al. A. Mickiewicza 30, 30-059 Krakow, Poland; 2 AGH University of Science and Technology, Faculty of Material Science and Ceramics, al. A. Mickiewicza 30, 30-059 Krakow, Poland; 3 University of Silesia, Faculty of Earth Sciences, ul. Będzińska 60, 41-200 Sosnowiec, Poland

**Keywords:** Geochronology, Radionuclides, Heavy metals, Bathymetry, Environmental changes

## Abstract

The Sudety Mts. form a chain of mountains in the South of Poland and during the last 200 years were subjected to strong industrial and agricultural pressure. The records of these human-induced changes are stored in natural archives like lake sediments. For the comprehensive study, three sediment cores taken from Mały Staw Lake (Sudety Mts.) were analyzed for the concentration of K, Na, Mn, Fe, Cu, Mg, Zn, Cd, Cr, Ni, Pb and radioactivity of ^137^Cs and ^210^Pb. As a result of the studies, the bathymetry map was developed and the sources of solid material supplied to the lake were identified. The geochronology studies of the cores were performed using ^210^Pb method, to evaluate model of time changes in the sediment. Radioactivity of ^210^Pb_uns_ (determined indirectly by ^210^Po) ranged from 1051 ± 64 to 12 ± 8 Bq kg^−1^. The ^137^Cs radioactivity was determined directly by gamma spectrometry and varied from 525 ± 37 Bq kg^−1^ for top layers to 9.80 ± 5.40 Bq kg^−1^ for the bottom of the core. Two characteristic peaks of ^137^Cs radioactivity related to the global fallouts after nuclear weapons testing and the Chernobyl accident were observed and used to confirm ^210^Pb dating method. Chemometrics analysis of the chosen metal’s concentrations combined with sample dating showed distinct imprint of human activity on the studied area.

## Introduction

Generally, the sediments consist of particles that have been transported by air, water, or glaciers from the sites of their origin in a terrestrial environment and have been deposited on the river, lake, or ocean bottom. The chemical composition of the sediments mainly depends on the geological structure, geomorphology, morphometry (concerning lakes), and climatic conditions. Undoubtedly, the location of the catchment or lake has an extensive impact on the sediments’ composition (Noges et al. 1999; Last and Smol 2001). Besides, sediments contain toxins including heavy metals, organic compounds, radionuclides, etc. So, they constitute natural repositories for different kind of contaminants and play important role in the distribution of toxic substances in the aquatic ecosystems (Sandor et al. 2001; de Deckere et al. 2011; Grba et al. 2016).

There are two main sources of toxins: natural, and those related to human activity. Radioactivity is an integral part of the environment. Radionuclides are found naturally in each part of the environment as water, soil, sediments, and air. Among natural radionuclides, ^210^Pb radioisotope can be distinguished. It is a long-lived (T_1/2_ = 22.3 years) radionuclide belonging to the uranium ^238^U radioactive chain (Appleby 2008). Special attention is attributed to the radionuclides which enter the environment in an uncontrolled way. In this respect, the distribution of ^137^Cs can be considered. This artificial radionuclide was introduced into the environment in a huge amount during nuclear weapon tests (1945–1965) and was emitted to the atmosphere during the Chernobyl accident (1986) (UNSCEAR 2000). Fallout from ^137^Cs and ^210^Pb have been widely used to establish the chronology of sediment cores collected from various aquatic systems (McCall et al. 1984; Ritchie and McHenry 1990; Walling and He 1992; Aycik et al. 2004; Klaminder et al. 2012; Szczepanska et al. 2012).

Considering the presence of metals (including heavy metals) in the environment, it should be noted that the natural cycle of trace elements is characterized by a certain balance between the amount of the element released by the geochemical processes taking place at the Earth’s surface, and the amount of element bounded in geological formations. In contrast, the elements emitted to the environment as a result of human activity (industry, transport, agriculture) form the risk of losing the chemical balance in biosphere, if introduced in excessive amount (Dube et al. 2001; Lofrano et al. 2016).

In most cases, the sediments’ sampling from the mountain lakes is challenging but their analysis forms valuable contribution to our knowledge of the environment, not only in local but also in regional scale. Especially, complex analysis of the sediments using various scientific approaches can provide interesting information about the water ecosystem. On one hand, sediments can be used for estimation of the state of the water ecosystem (Teuchies et al. 2011; Guzman et al. 2013; Hamerlik et al. 2016). On the other hand, they are often used for studying history of environmental contamination as well as climate and environmental changes (Smol et al. 1991; Bitusik et al. 2009; Ma et al. 2013; Szarlowicz et al. 2013).

In this study, the analytical, radiochemical, sedimentological, and geomorphological methods were used in order to determine changes of the sediments collected from the Mały Staw Lake (Sudetes). The Mały Staw Lake (50°45′N, 15°42′E) is interesting due to the following reasons: it is localized in the unique Sudety mountains and surrounded by the steep slopes of over 200 m high; hydrological and meteorological conditions in the area are well defined; reference information concerning geology, composition and properties of rocks and soils, as well as human activity in the past 200 years is also well documented; and last but not least—no such research have ever been undertaken. The aims of the study included determination of the following: the bathymetric survey, identification of the potential sources of material transported to the lake, estimation of the age and sedimentation rate of each layer of the sediments’ cores by use of the ^210^Pb and ^137^Cs methods, and determination of the concentration of chosen metals with identification of their origin.

## Study area—the Sudetes and the Mały Staw Lake—general information

**Fig. 1:**
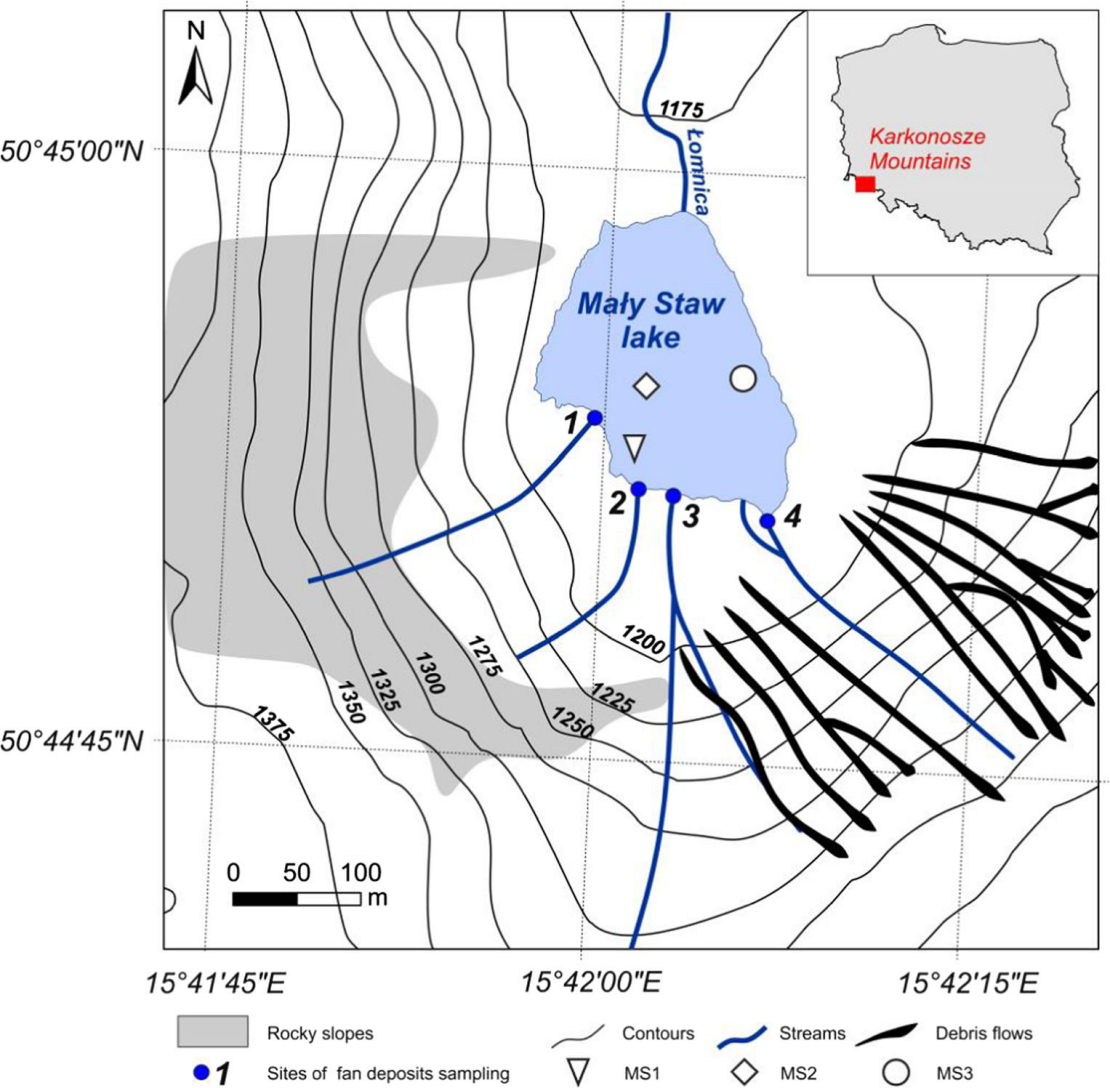
Location of the studied lake

The Sudety mountain range is the highest part of the Czech Massif. It stretches from eastern Germany along the northern border of the Czech Republic to south-western Poland. The Sudetes are a typical example of the horst mountains and their geological structure is highly diversified. The highest part of this old and eroded chain of mountains is the Karkonosze Mts., reaching 1602 m a.s.l. at Mt. Snieżka. The Karkonosze massif is built mainly of granites in which the Mały Staw cirque was created in Pleistocene. The Mały Staw Lake is a cirque, post-glacial lake and it is situated in the Polish Karkonosze National Park (Fig. 1). The bottom of the Mały Staw lake lies at an altitude of 1183 m a.s.l. The lake has area of 2.8 ha and 756 m of coastline. The Mały Staw Lake is a flow-through lake, drained by Lomnica River, and the Mały Staw cirque is surrounded by steep granite walls of Smogornia and Snieżka massifs. The surrounding 200 m high walls are cut by few couloirs. Weathered granites of the Karkonosze create gravely and sandy grains, which are easily transported down by gravitation, water, avalanches, or debris flows. The weathered mineral material in favorable conditions creates a debris flows. There are 16 active debris flows in the Mały Staw cirque but none of them reaches the Lake (Jahn 1985; Parzoch et al. 2007). Some valuable information about the historical changes of the lake biota were determined by means of paleobiological research. These revealed, despite climatic factors, also influence of chemical factors like strong dependence of life in the lake on the water body pH (Sienkiewicz et al. 2006).

## Methods

### Bathymetry

The depth of Mały Staw Lake was measured by means of Lowrance HDR-5 with a 50/200-kHz transducer. The Lowrance depth sounder is equipped with a built-in GPS which allows for the logging of depth and co-ordinate data at the same time. Bottom depth measurements were taken multiple times per second along the logging paths. The obtained data set is composed of depth measurements and the associated GPS co-ordinates. The final map was generated by means of ArcGis instruments. The bathymetric map is based on depth points from echo sounder measurements. They were interpolated to produce a quantitative map using the Inverse Distance Weighting (IDW) method.

### Sampling

The sediments’ samples were collected during scientific survey in 2013. Three sediments’ cores were sampled using Limnos gravity corer (sampler consisted of a set of 1 cm high rings of 10 cm diameter). Sediments’ cores were collected at three different depths: MS1-2 m (15°42′1.5″E 50°44′53.3″N), MS2-5 m (15°42′1.8″E 50°44′54.6″N) MS3-3 m (15°42′5.9″E 50°44′54.9″N) (Fig. 1). The cores were sliced to 1 cm layers in situ, and packed into polyethylene vessels. The top sediments’ layers were of low density, while moving to the bottom layers of the core, density increased.

The sediment samples were also taken from four alluvial fans entering the Mały Staw Lake (Fig. 1).

### Granulometric analysis of fans deposits

The samples of sediments originating from the weathering granite massif surrounding the lake were taken from four alluvial fans entering the Mały Staw Lake. Samples were air-dried before the granulometric analysis. To determine the percentage of different grain sizes, the granulometric analysis of sediments was performed involving a nested column of sieves placed in a mechanical shaker. For separation of fractions, the sieves with openings of 8, 4, 2, 1, 0.5, 0.25, 0.125, and 0.063 mm were used.

### ^210^Pb and ^137^Cs chronologies

The age of sediments was estimated for the deepest MS-2 core. In this sediment’s core, the metals’ concentrations were also determined. In all sediments’ cores, the distribution of cesium radioactivity was measured. Such measurements do not require laborious radiochemical procedure and are valuable in interpretation of the scale of artificial radionuclide contamination.

Dating of sediment was established by applying the Constant Rate of Supplying (CRS) model to the measured ^210^Pb_uns_ data (Appleby and Oldfield 1978; Appleby 2001). The radioactivity of unsupported ^210^Pb was calculated from total radioactivity of ^210^Pb by subtraction of the supported radioactivity. The supported radioactivity was determined by measurements made on old sediments. Radioactivity of the sediments in the deepest part of the core is ascribed to the supported ^210^Pb radioactivity (the oldest sediments contain no unsupported ^210^Pb). It uses the equation presented in Szarlowicz et al. (2013) and the age and the sedimentation rate of each layer were estimated. Usually ^210^Pb dating should be confirmed by another method, so ^137^Cs was used as a tool for ^210^Pb validation. As the presence of ^137^Cs at elevated levels was known to occurred during 1963 (maximum of global fallout) and 1986 (fallout of Chernobyl accident), the observed maxima in ^137^Cs level in the sediment cores could have been ascribed to these incidents and correlated with the depth of sediment (Klaminder et al. 2012).

### ^210^Pb measurement

**Fig. 2:**
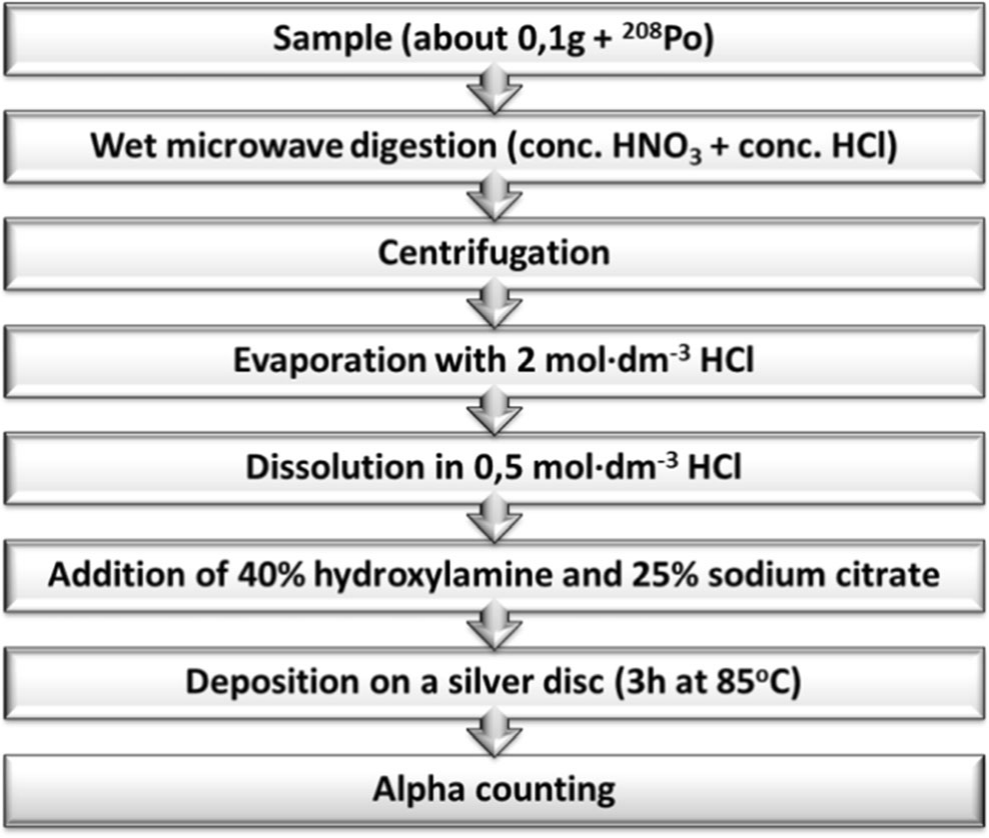
Scheme of radiochemical procedure of polonium determination

To determine lead, the alpha spectrometry method was used. Alpha spectrometry requires radionuclides separation. ^210^Pb_tot_ in the sediment samples was determined by carrying out a spontaneous deposition of ^210^Po (daughter radionuclide in radioactive equilibrium with its parent ^210^Pb) on the surface of a silver disc (Flynn 1968). First, polonium in all analyzed samples was determined. The alpha sources were measured using an alpha spectrometer (Canberra model 7401, equipped with Passivated Implanted Planar Silicon (PIPS) detector active area 450 mm^2^, efficiency 34%). A single measurement lasted 3 days. After about 6 months, a sufficient amount of new ^210^Po had been grown in the sample; a new deposition was carried out. In Fig. 2, the scheme of the procedure is shown. Generally, the procedure consists of the following: sample digestion, evaporation, sample preparation for deposition, deposition. All analyses were carried out with ^208^Po, a radiotracer used to control efficiency of the procedure. Known radioactivity of polonium from two depositions enabled calculation of lead total radioactivity (Szarlowicz et al. 2013).

### ^137^Cs measurement

The sediment core samples were analyzed for ^137^Cs (T_1/2_ = 30.07 years) at 661.6 keV by means of gamma spectrometry. Gamma measurement was conducted on a low background, high purity coaxial germanium detector (Canberra, model GC2020, relative efficiency 20%) coupled with a multi-channel analyzer. The counting system was calibrated for energy and efficiency. The energy calibration was carried out with known radionuclides of varying gamma energies (Spectrum Techniques, ^133^Ba, ^137^Cs, ^60^Co, ^65^Zn). The efficiency calibration was done using the reference material (IAEA-447) supplied by the International Atomic Energy Agency (IAEA, Vienna, Austria) according the procedure proposed by Misiak et al. (2011). The samples were measured in the standardized polyethylene vessels (volume 120 cm^3^, cylindrical—5 cm diameter and 7 cm high), for 3 days. All radioactivity measurements reported, refer to dry mass of the sample, and were calculated for the day of sampling.

### Heavy metals determination

Quantitative determination of metals in the sediments core samples was performed after the following sample preparation procedure. The samples were air-dried, sieved, powdered, and homogenized. Then, the samples were wet digested (the single sample weight was about 0.1 g) using the mixture of nitric acid (65%) and hydrogen peroxide (30%) (Suprapure, Merck, Germany) in an Anton Paar Multiwave 3000 (Switzerland) microwave system. After digestion, the volume of reagents was reduced by evaporation on a hot plate and the solution was filtered into a 10-ml volumetric flask. Simultaneously, the blank samples were prepared to control possible samples contamination. Each of the processed sediments’ sample was digested in two replicates.

Sodium and potassium concentrations were determined using the flame photometry method (AES), copper, magnesium, iron, manganese, and zinc were determined by means of the flame technique of atomic absorption spectrometry method (F AAS), while nickel, cadmium, lead, and chromium by means of the electrothermal AAS. Analyses were performed using the Perkin Elmer spectrometer Model 3110 (AES, F AAS, USA) equipped with the graphite furnace Perkin Elmer HGA 600 (ET AAS, USA) and Perkin Elmer spectrometer Model 4100 ZL (Germany) (used in Cr and Ni determinations). The analytical procedure was optimized in respect to the sample preparation and quantitative determination of the elements. In Table 1, the techniques used and basic instrumental parameters are listed. Accuracy of the determinations was checked by analysis of the certified reference material, River Sediment CRM 320 (the analytes—Cd, Cr, Cu, Ni, Pb, Zn). Good agreement was found in respect to the elements’ certified concentration values.

**Table 1:** Instrumental parameters of the elements quantitative determination

Element	Technique	Optical parameters		Description
		Lamp type	Wavelength [nm]	
K	AES	HCL	766.5	Air-acetylene flame, standard conditions
Na	AES	HCL	589.0	Air-acetylene flame, standard conditions
Cu	F AAS	HCL	324.8	Air-acetylene flame, standard conditions
Fe	F AAS	HCL	248.3	Air-acetylene flame, standard conditions
Mg	F AAS	HCL	285.2	Air-acetylene flame, standard conditions
Mn	F AAS	HCL	279.5	Air-acetylene flame, standard conditions
Zn	F AAS	HCL	213.9	Air-acetylene flame, standard conditions
Cd	ET AAS	EDL	228.8	Pyro/platform tube, Deuterium Lamp background correction
Cr	ET AAS	HCL	357.9	Pyro/platform tube, Zeeman background correction
Ni	ET AAS	HCL	232.0	Pyro/platform tube, Zeeman background correction
Pb	ET AAS	EDL	283.3	Pyro/platform tube, Deuterium Lamp background correction

## Results and discussion

**Fig. 3:**
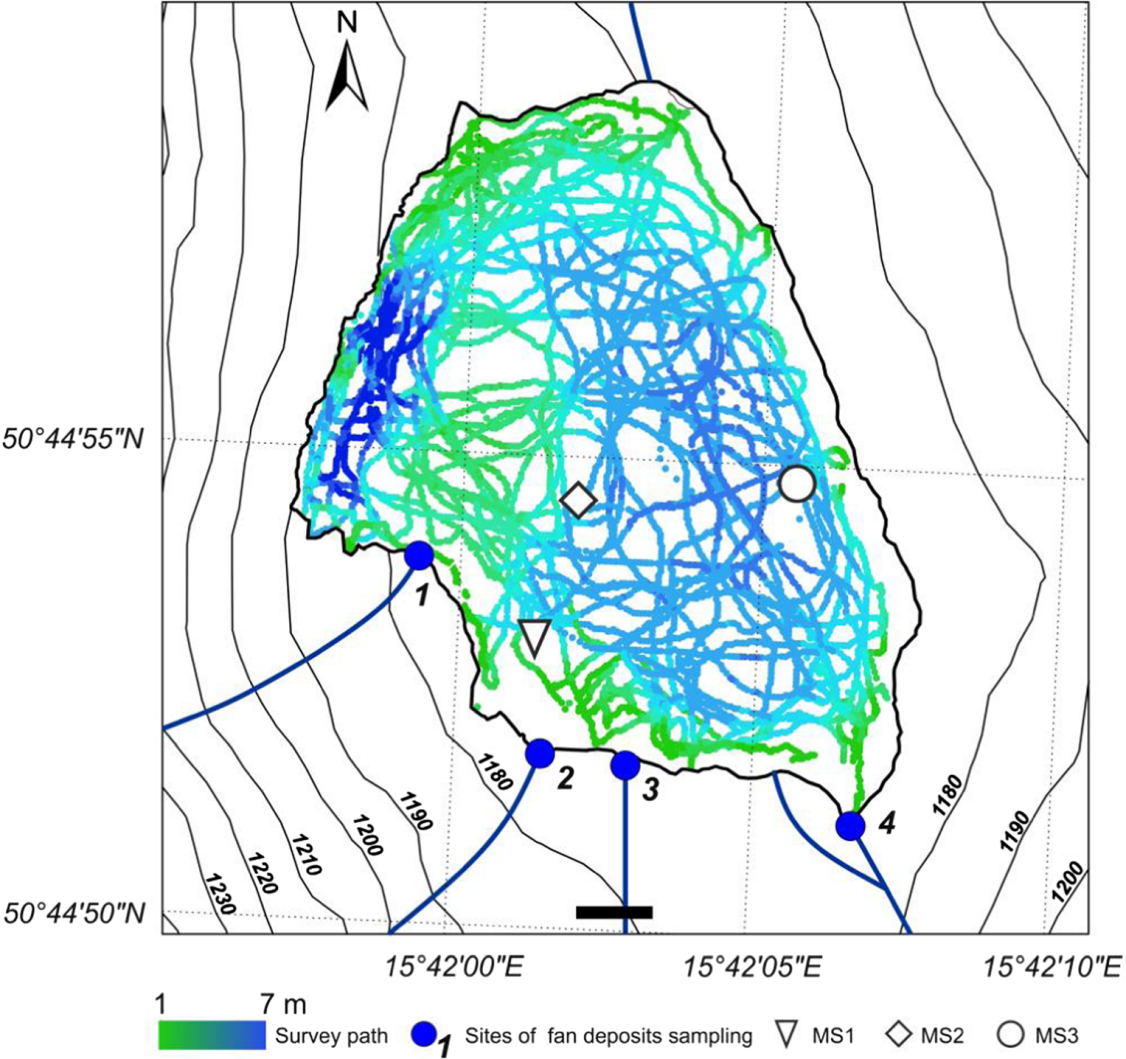
Map showing locations of track lines along which depth data were collected

The bathymetric survey provided a detailed map of the Mały Staw Lake floor topography. Track lines along which the collected depth data are presented in Fig. 3. Each point in the map reflects one measurement point in the field. The maximum depth, located in the west part of the Lake, is 6.93 m. A similar value was given by Komar (1949).

The average depth of the lake is 3.4 m and the lake bottom is divided into two basins: the deeper, narrow west basin and the shallower west basin. We did not examine the character of the ridge dividing the lake. According to the previous research, it may be a remnant of moraine (Piasecki 1958).

**Fig. 4:**
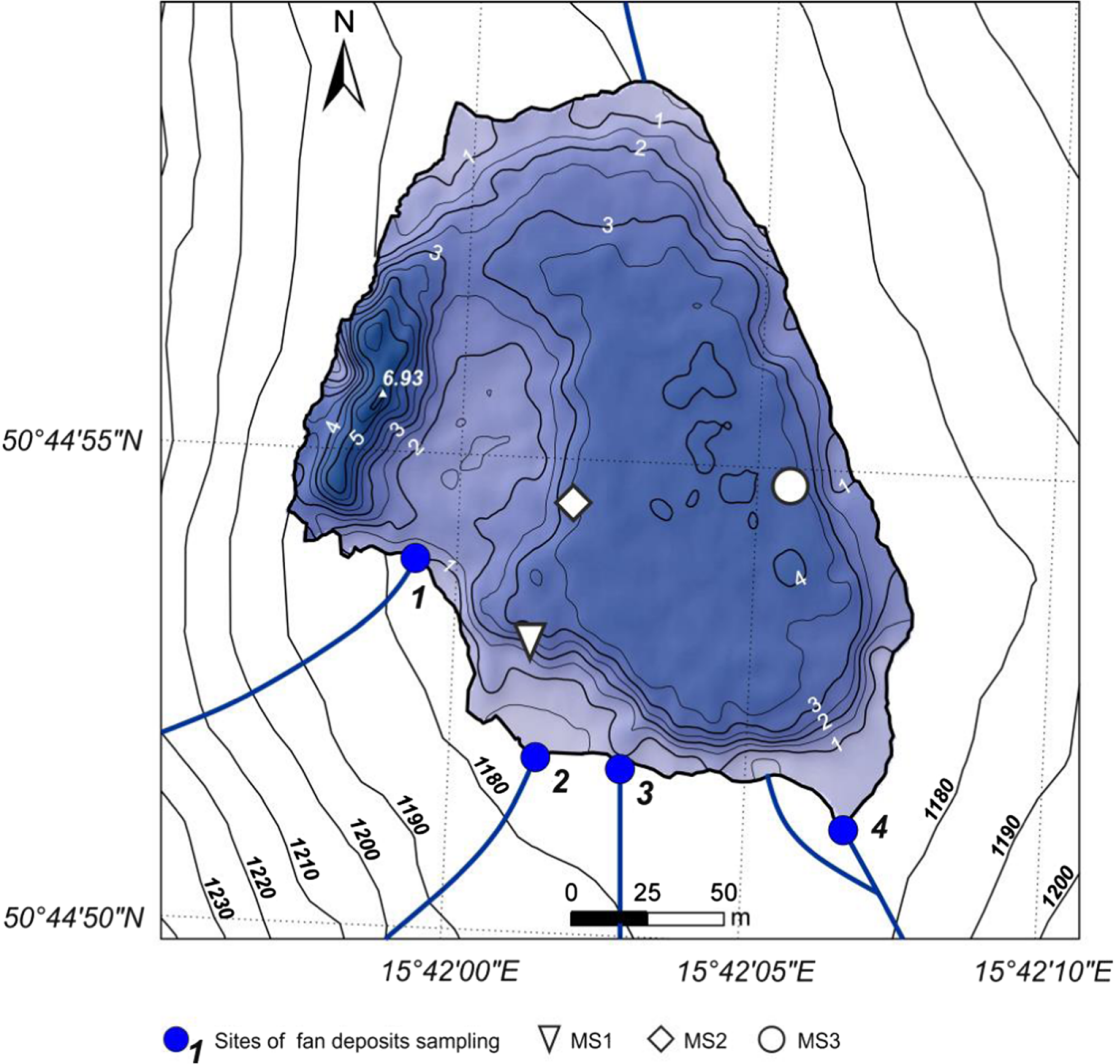
Bathymetric map of the Mały Staw Lake

Along the south shore, four alluvial cones are clearly visible in the plotted bathymetric map (Fig. 4). Fans 1, 2, and 4 go under the water lake to the depths of approximately 1.5, 3.5, and 2 m, respectively. Most deposits of fan number 3 are deposited at the land, as the slope of the last reach of the stream transporting the material is relatively low.

**Fig. 5:**
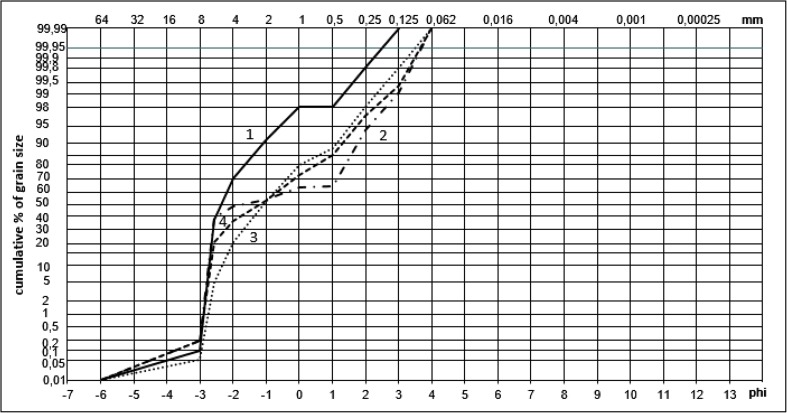
Granulometric curves of alluvial fans deposits

The sediments of debris flows are poorly sorted and the finer material is periodically washed out of the debris flow deposits by water from melting snow or from precipitation. This material creates alluvial fans partially entering the lake and the finest grains are deposited at the lake bottom. Łomnica is the only permanent (except in winter) watercourse supplying the Mały Staw Lake with water and sediments. In Fig. 5, the granulometric curves of alluvial fans deposits are shown. Fan number 1 consists mainly of gravel, and numbers 2, 3, and 4 of sandy gravel. Deposits of fan number 2 are poorly sorted (standard deviation = 2.25) while deposits of fans 1, 3, and 4 are better sorted (standard deviation is 1.07, 1.36, and 1.85, respectively). The reason for these differences may be the fact that fan number 2 is created by weathered material, transported down by the shortest stream among four discussed, which creates less possibility to sort the fractions during transportation.

**Fig. 6:**
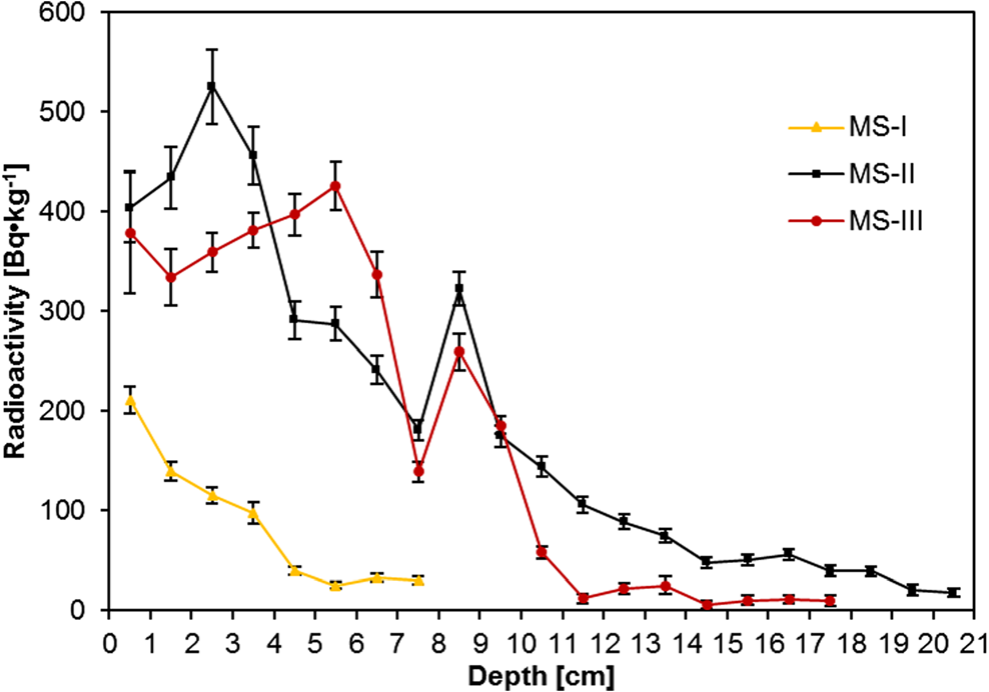
Changes of ^137^Cs radioactivity in the sediment cores

The ^137^Cs radioactivity was measured and varied in the range from 9.80 ± 5.40 to 525 ± 37 Bq kg^−1^. Vertical distribution of ^137^Cs in the sediment cores of the Mały Staw Lake is presented in Fig. 6. Despite the fact that the sampling points were located in different regions of the lake (and also differing in depth), the ^137^Cs distribution is quite similar. In each sediment core, cesium radioactivity is at the highest level in the uppermost sediment layer and decreases with depth down the core. The highest value of ^137^Cs radioactivity in the top layer of the sediments relates to delivery of this radionuclide from the soil surrounding lake and Łomnica slopes. The shortest of the sediment cores is different comparing to the other two. This sediment core was located in the shallow part of the reservoir where the coarse grain size material and stones are deposited. Such conditions do not favor ^137^Cs sorption. Besides, this sediment core is too short to interpret distribution of this radionuclide. In two longer sediment cores, ^137^Cs radioactivity declines no monotonously with two distinct maxima corresponding to depth ranges 4.5–5.5 and 8.5–9.5 cm. The elevated level of the ^137^Cs radioactivity corresponds to the nuclear weapon tests (1963) and the Chernobyl accident (1986) (UNSCEAR 2000). Firstly, the fallout from nuclear weapons tests had an influence on the radioactive contamination level of the environment in Poland. But the main contribution to the growth of the radioactive contamination is ascribed to the Chernobyl accident. Fission products such as ^137^Cs were moving with the masses of air according to actual meteorological conditions and then they were depositing on the surface. The highest levels that are observed in the South of Poland are the result of intensive local rainfall occurring in these areas during the Chernobyl accident (Biernacka et al. 2004; Kardas et al. 2011; Isajenko et al. 2012). The obtained results confirmed data collected by a meteorological station located at the Snieżka Mt. Distinct ^137^Cs radioactivity maxima (Fig. 6), observed between 1959 and 1965 and in 1986, resulted from the aforementioned events (Czerwinski 1995). The cesium radioactivity still decreases as a results of its natural radioactive decay.

**Fig. 7:**
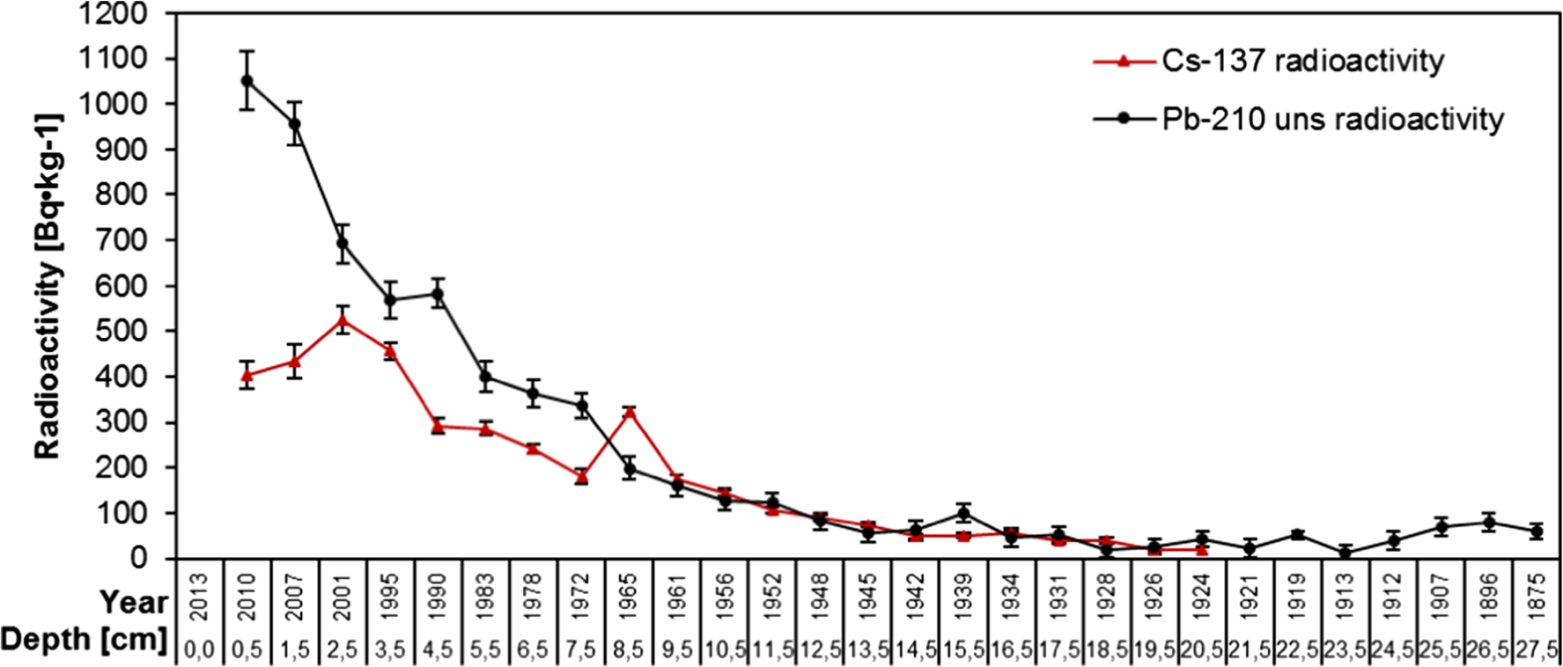
Depth-age model based on ^210^Pb and ^137^Cs geochronology

The profile of unsupported ^210^Pb concentration for the MS2 core is shown in Fig. 7. Radioactivity of ^210^Pb_uns_ was in the range of 12 ± 8 to 1051 ± 64 Bq kg^−1^. Regular decrease of radioactivity of this radionuclide in the discussed sediment core was observed. The radioactivity declines in a stepwise manner. There are some irregularities from 17.5 cm down the sediment core. Probably, some disturbances and/or sediment mixing occurred. The CRS model was used and the sediment core was dated. The lead dating indicates that 28 cm of the core represents the past ca 138 years. The results from ^210^Pb dating were confirmed by ^137^Cs studies. Sediments in the depth range 4.5–5.5 cm correspond to the period 1983–1990 (Chernobyl accident) and those at depths ranging between 8.5 and 9.5 cm were assigned to the years 1961–1965 (nuclear tests) (Fig. 7).

**Fig. 8:**
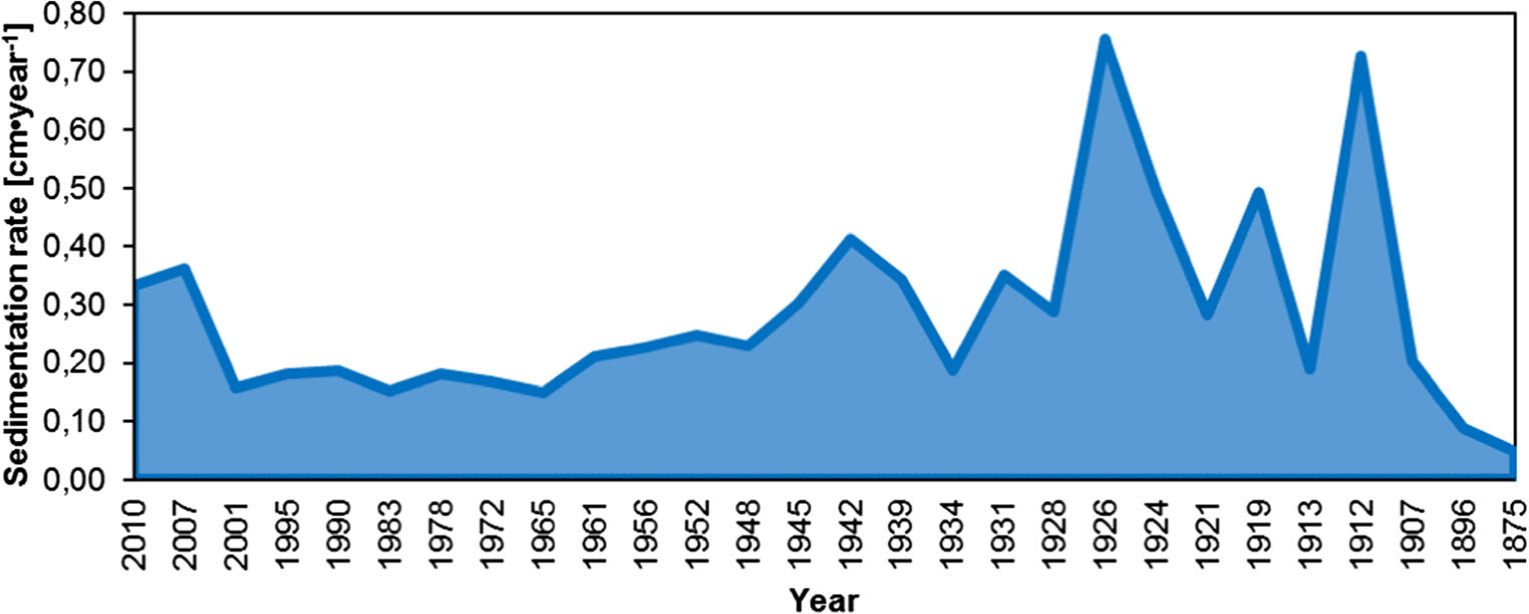
Changes of sedimentation rate in the studied period of time

The deposition time of each sediment layer along the core was different. In the first two layers, it was around 3 years, going to third layer (2001), it was 6 years for 1 cm. Next, in the second half of the twentieth century, this time was estimated between 4 and 7 years for each 1 cm layer. Going deeper, from 13 cm (1948) to 25 cm (around 1912), deposition time was estimated to be around 3 years per 1 cm layer. Finally, from 26 cm (1907) to 28 cm (1875), it was 5, 11, and 21 years per 1 cm layer, respectively. The length of deposition time is reflected in the values of the sedimentation rate (Fig. 8). The shorter the deposition time, the higher is the value of sedimentation rate. The average sedimentation rate of the core was estimated to 0.28 cm/year. There can be indicated periods for which the higher values of sedimentation rate were distinguished. The first one, from 2007 to 2010, corresponded to the period of extraordinary rainfall (the fallout was at the highest level since 2000). In total, 1272–1316 mm of precipitation fall in the studied area (CSO 2008, 2009, 2010, 2011). This resulted in transport of high amounts of solid material, for example with storm water, down the slopes of the surrounding mountains. Considering beginning of the twentieth century (up to 1939), the values of sedimentation rate were at their highest level. This can be explained by the delivery of a huge amount of deposited material in a short time induced by, e.g., avalanches, meteorological situation, or melting snow. The historical records show that in this period of time, rainfall was rather at average level, but in 1913 a heavy rainfall event occurred. Following this, numerous avalanches also occurred and the biggest in the Mały Staw cirque was reported in 1928 (Jahn 1985).

For the first time, in respect to the sediments of the Mały Staw Lake, the chosen metals’ concentrations in the collected sediments core samples were determined (Tables 2 and 3). Concentrations of potassium, magnesium, iron, sodium, and manganese were the lowest in the top 1 cm layer of the sediments’ core. The highest concentration of K was in the layer at 22 cm (dated to the year 1921) while magnesium in the layer at 16 cm deep (year 1939). In the layer adjacent to the top one, concentration of sodium was the highest. Considering concentrations of iron and manganese, highest concentrations were found in the layers between 26 and 30 cm of the core (Table 2).

**Table 2:** Concentrations of K, Mg, Fe, Mn, and Na in the sediments’ core samples of the Mały Staw Lake in reference to the layer depth

Sample [cm]	Age [years]	K	Mg	Fe	Mn	Na
		[μg g^−1^] ± SD				
1	2010	1022 ± 5	1432 ± 11	8027 ± 64	90 ± 1	79 ± 1
2	2007	3649 ± 240	1925 ± 96	9977 ± 548	108 ± 7	237 ± 7
4	1995	3853 ± 16	2076 ± 48	10,535 ± 428	118 ± 2	221 ± 1
6	1983	3823 ± 117	2147 ± 32	10,683 ± 256	117 ± 3	235 ± 40
8	1972	3727 ± 122	2212 ± 43	10,810 ± 156	114 ± 3	202 ± 20
10	1961	3532 ± 189	2325 ± 25	11,205 ± 219	120 ± 3	197 ± 54
12	1952	3911 ± 110	2530 ± 204	12,248 ± 128	129 ± 2	195 ± 8
14	1945	3835 ± 145	2595 ± 45	11,955 ± 556	130 ± 2	159 ± 4
16	1939	4152 ± 152	3315 ± 19	12,988 ± 1315	138 ± 2	184 ± 3
18	1931	3798 ± 19	2829 ± 31	12,075 ± 116	134 ± 2	151 ± 13
20	1926	3258 ± 13	2942 ± 8	13,090 ± 198	126 ± 1	127 ± 1
22	1921	4526 ± 113	2704 ± 74	12,960 ± 1580	144 ± 1	202 ± 26
24	1913	4037 ± 250	2744 ± 117	9338 ± 2158	138 ± 2	160 ± 20
26	1907	4265 ± 348	2718 ± 108	13,415 ± 1558	143 ± 4	166 ± 32
28	1875	4301 ± 204	2590 ± 101	12,033 ± 575	140 ± 1	172 ± 15
30	–	4185 ± 497	2644 ± 152	11,253 ± 283	145 ± 6	156 ± 33
32	–	3831 ± 339	2341 ± 21	9940 ± 106	120 ± 2	137 ± 22
33	–	3540 ± 399	2359 ± 66	9923 ± 332	124 ± 2	117 ± 13

**Table 3:** Concentrations of Pb, Zn, Cd, Cu, Cr, and Ni in the sediments’ core samples of the Mały Staw Lake in reference to the layer depth

Sample depth [cm]	Age [years]	Pb	Zn	Cd	Cu	Cr	Ni
		[μg g^−1^] ± SD					
1	2010	206 ± 1	81.10 ± 0.50	16.10 ± 0.60	19.2 ± 1.3	12.20 ± 0.50	5.50 ± 0.40
2	2007	222 ± 14	85.6 ± 7.5	26.1 ± 2.8	24.3 ± 1.8	19.50 ± 0.40	7.56 ± 0.85
4	1995	216 ± 4	90.3 ± 1.5	13.00 ± 0.70	27.2 ± 0.60	20.00 ± 0.30	8.00 ± 0.49
6	1983	224 ± 14	100.70 ± 0.50	15.1 ± 4.2	27.20 ± 0.60	19.70 ± 1.2	8.93 ± 0.31
8	1972	210 ± 2	108.60 ± 0.90	18.70 ± 0.20	25.50 ± 0.50	19.00 ± 0.40	9.28 ± 0.97
10	1961	208 ± 17	105.3 ± 1.5	21.60 ± 0.09	24.00 ± 0.30	16.10 ± 0.60	7.62 ± 0.63
12	1952	201 ± 8	98.6 ± 1.6	17.2 ± 1.5	24.00 ± 0.80	17.4 ± 1.5	7.16 ± 0.17
14	1945	163 ± 2	82.7 ± 1.1	13.3 ± 2.8	22.40 ± 0.30	15.00 ± 0.30	6.70 ± 0.33
16	1939	154 ± 2	71.21 ± 0.62	5.72 ± 0.41	23.10 ± 0.32	15.70 ± 0.90	6.49 ± 0.32
18	1931	155 ± 2	72.6 ± 1.7	9.8 ± 0.2	23.01 ± 0.70	13.80 ± 0.21	6.21 ± 0.54
20	1926	151 ± 2	61.51 ± 0.40	9.30 ± 0.11	19.7 ± 2.3	12.50 ± 0.61	5.64 ± 0.49
22	1921	161 ± 10	65.7 ± 0.8	8.0 ± 0.4	22.8 ± 0.3	15.41 ± 0.60	6.92 ± 0.32
24	1913	150 ± 6	60.40 ± 0.61	8.11 ± 0.40	22.20 ± 0.41	13.2 ± 1.0	6.36 ± 0.03
26	1907	160 ± 1	67.10 ± 5.31	7.6 ± 1.7	22.90 ± 0.31	14.32 ± 0.60	6.66 ± 0.19
28	1875	153 ± 3	58.20 ± 0.51	7.20 ± 0.80	22.50 ± 0.70	14.0 ± 1.4	6.01 ± 0.19
30	–	158 ± 4	59.7 ± 1.7	7.6 ± 0.2	22.60 ± 1.01	13.6 ± 1.5	5.75 ± 0.25
32	–	138 ± 4	52.4 ± 0.8	7.7 ± 0.3	18.31 ± 0.40	12.01 ± 0.81	5.310 ± 0.070
33	–	138 ± 11	50.60 ± 0.81	7.2 ± 1.1	18.06 ± 0.10	11.00 ± 0.40	5.17 ± 0.14

Regarding concentrations of the other metals, it can be observed that the lowest concentrations of lead, zinc, cadmium, copper, chromium, and nickel were determined in the deepest, oldest layers of the core (depth 32–33 cm), the highest in much younger layers dated to years 1972–2007 (layers 2–8 cm) (Table 3).

To get the general view on the results concerning metals concentrations in the Mały Staw Lake sediments, some basic statistical analysis of the obtained results is summarized in Table 4.

**Table 4:** Concentrations ranges, mean values of the analyzed metals concentrations in the core samples of the Mały Staw Lake [μg g^−1^]

Element	Minimal concentration	Maximal concentration	Mean
Fe	8027	13,415	11,288
K	1022	4526	3830
Mg	1432	3315	2485
Na	79	237	176
Mn	90	145	128
Ni	5.17	9.28	6.81

Sediments contamination degree in general can be classified according to the toxic elements’ concentrations into three classes I—clean, II—moderately contaminated, and III—contaminated (Table 5). Also, the sediments quality can be discussed by comparing the obtained specific data to the natural concentration of the elements in the sediments (geochemical background for the Polish lakes’ sediments) (Bojakowska and Sokołowska 1998). Out of the five listed elements, only concentration of zinc is close to the geochemical background for Poland. Concentrations of the other heavy metals are substantially higher. On the other hand, if one considers the presented classification, depending on the element, the sediments of the Mały Staw Lake belong to the I^st^ class—sediments not contaminated (Cr, Cu, and Zn) or to the III^rd^ class, if Cd and Pb concentrations are discussed. Undoubtedly, the latter two elements high concentrations result not only from human activity but also from natural weathering of the mother rock, as the lowest determined concentrations (deepest sediments’ layers) were still several times (around 10 times) higher than the geochemical background for Poland.

**Table 5:** Comparison of the heavy metals’ concentrations in the Mały Staw Lake with geochemical background and contamination classification. Concentration values in milligram per kilogram

Element	Mały Staw Lake (mean)	Mały Staw Lake (maximum)	Geochemical background	Contamination classification		
				I class	II class	III class
Cd	12,5	26.1	< 0.5	1.0	3.5	6
Cr	15.4	20	6	50	100	400
Cu	22.9	27.2	7	40	100	300
Pb	176	222	15	30	100	200
Zn	76.5	108.6	73	200	600	1000

Generally, Karkonosze Mts. ridge is built of Paleozoic granitic rocks and slate metamorphic rocks. It causes that the soils of Karkonosze reveal fragmentary characteristic of specific properties. pH of the Karkonosze soils is acidic, below 5.0 with the lowest values of 3.5, which is their genetic property and only to a little extend it is influenced by pollution of the region (acid rains) (Skiba 1995). For the sorptive properties of the prevailing in Karkonosze mineral soils, considering small amount of clay minerals in the soils, mainly carious material is responsible (Kabala 2011). Both these factors cause that metals delivered to the soil in the result of industrial pollution (which can be attributed to the so called “screen effect of mountainous systems”) can easily migrate between the environment components (soil, water, sediments). Taking this into account and comparing the obtained results with data reporting concentration of heavy metals in the surface layers of soils of the Karkonosze (Skiba et al. 1994; Skiba 1995), meaningful similarities to the sediments’ compositions can be found. For the Snieżka massif, concentrations of Cr, Cu, Pb, and Zn were in the same order of magnitude as determined in the sediments of the Mały Staw Lake (Table 5). These were as follows (ranges of concentrations in mg kg^−1^): Cd 1.69–3.88; Cr 6.94–22.3; Cu 6.54–38.19; Pb 91.4–312.5; Zn 12.4–94.5 (Skiba et al. 1994). This fact proves mobility of the elements resulting from their chemical properties (for example Cd and Zn), and/or mobility with the sorbent (elements adsorbed by carious material—Pb, Cu, Ni, Cd) (Binczycki et al. 2015).

**Fig. 9:**
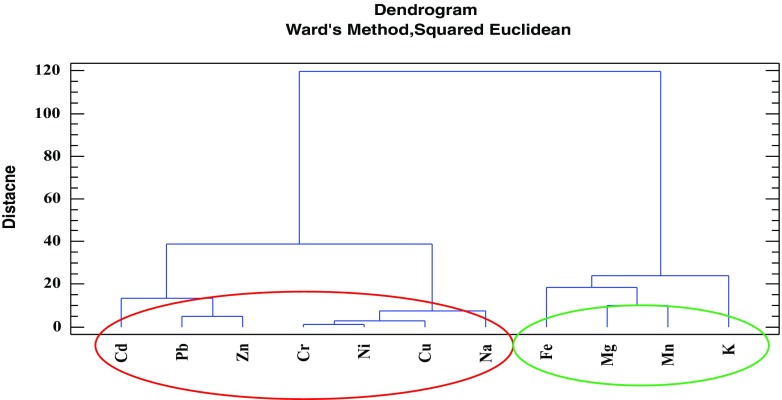
Dendrogram presenting similarity of the elements’ concentrations variability in the sediments core of the Mały Staw Lake

**Fig. 10:**
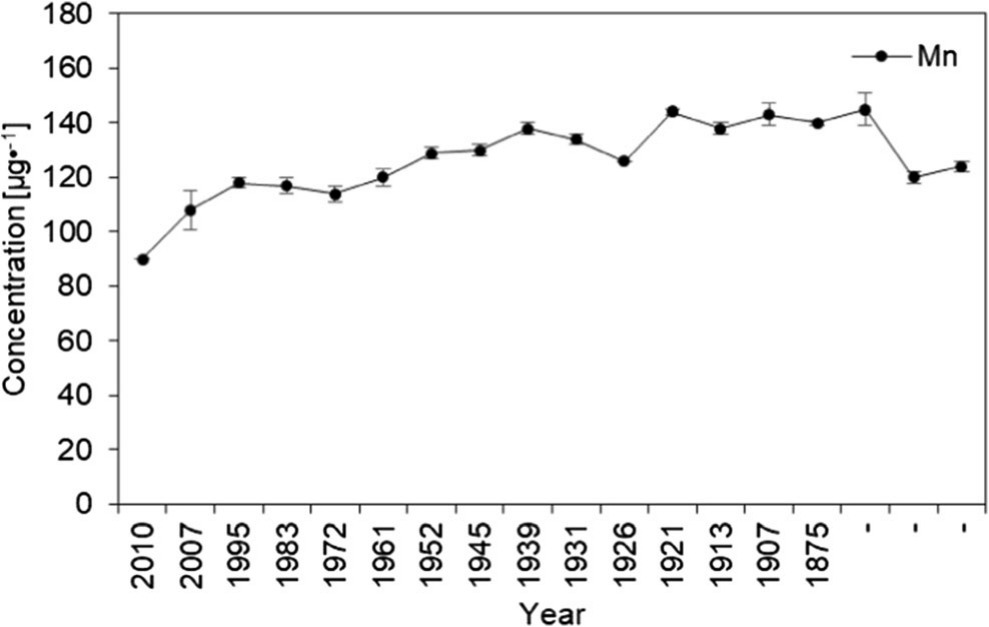
Changes of manganese concentration in the sediments core of the Mały Staw Lake

**Fig. 11:**
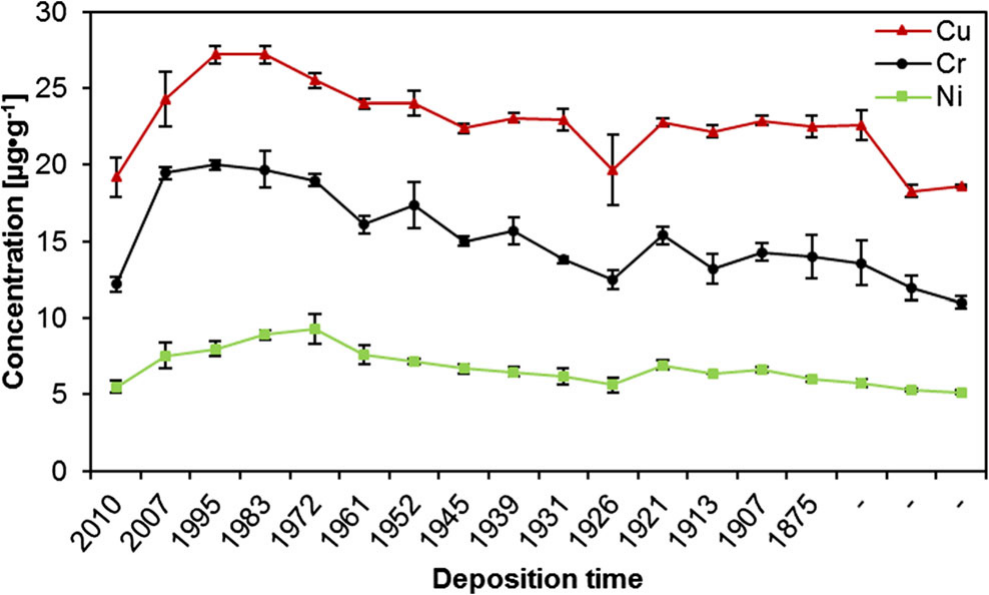
Changes of copper, chromium, and nickel concentrations in the sediments core of the Mały Staw Lake

**Fig. 12:**
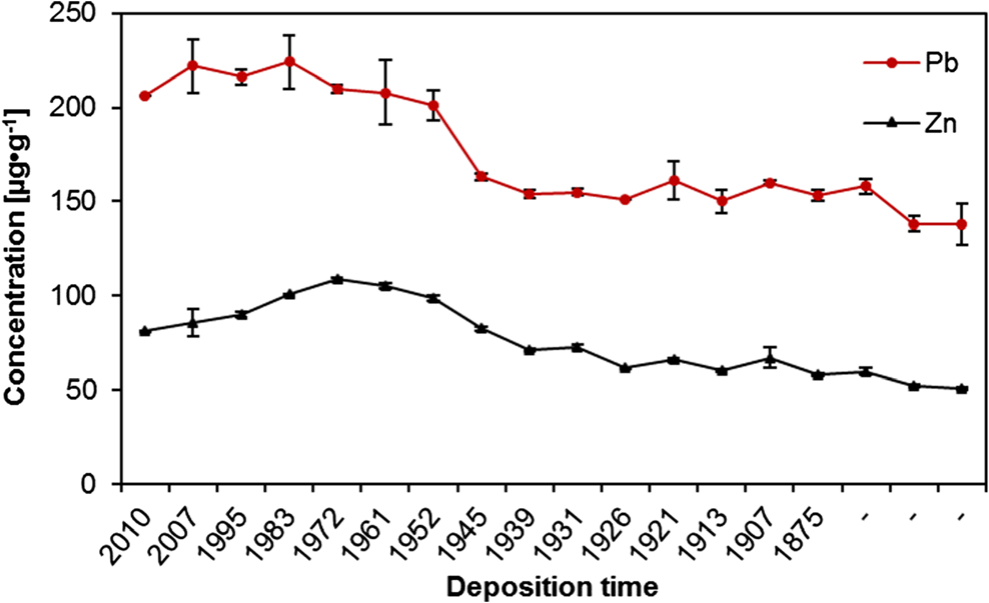
Changes of lead and zinc concentrations in the sediments core of the Mały Staw Lake

The obtained quantitative data can be discussed basing on the use of certain chemometric tools, enabling multidimensional analysis. To extract meaningful relationships between the analyzed objects (sediments’ core layers) and variables (elements concentrations), cluster analysis (CA) and principal components analysis (PCA) were chosen. All elements concentrations values were standardized prior to chemometric analysis. Considering groups of the elements (Fig. 9), two main clusters can be distinguished. The first one, marked with the green oval, consists of Fe, Mn, Mg, and K—the elements which are present in the soil and rocks of Karkonosze naturally and in relatively high concentrations (Skiba et al. 1994; Skiba 1995; Binczycki et al. 2015). Their changes in the core are similar and may be influenced mainly by the natural climatic and weathering processes. Human activity has lesser influence on the elements transport and accumulation in the sediments in time. All four elements are characterized by small concentration variability and similar course of changes (with the exception of the first, top sediments layer) (Table 3 and Fig. 10). The second main cluster (marked with the red oval) (Fig. 9) consists of trace metals and sodium. In this cluster, two sub-clusters are visible. One, consisting of Cr, Ni, Cu, and Na, represents elements showing almost constant increasing tendency of the elements’ concentrations in time (Fig. 11), while the second sub-cluster (consisting of Pb, Cd, and Zn) is characterized by slight different course of the elements concentration changes—initial low concentration in the deep sediments layers, substantial increase in the layers dated back to the second half of the twentieth century, and decrease of concentrations in recent years (Fig. 12). Definitely, this increase of the elements concentrations results from human industrial activity in the discussed area but also results from the deposition of pollutants transported in the atmosphere to the long distances and as a result of acidity of the Karkonosze soils, promoting washing out of the adsorbed elements from the soil surrounding the lake. The Karkonosze Mts. form the natural barrier for the wind. Air masses predominantly (75%) flow from the industrialized regions of the Czech Republic (Most-Teplice), Germany (Cottbus), and Poland (Turoszów, Gubin, Jelenia Góra). They are characterized by the elevated levels of heavy metals content, and due to dry and wet deposition are the source of soil and water contamination in the region.

The decrease of the elements concentrations in the twenty-first century is a direct result of introduction of new measures of environment protection in Europe and Poland. It is worth to notice that considering concentrations of Cr, Cu, and Ni in the sediments, their current level (in the top layer of the sediments) is comparable to the level found in the deepest layers, what suggests that currently their concentration is mainly influenced by the natural processes. On the other hand, concentrations of Pb, Cd, and Zn are still substantially higher in the younger layers than in the old ones—these elements are constantly delivered to the environment of the Mały Staw Lake (Fig. 12).

It is worth to add that increasing touristic activity could influence quality of the sediments (Mierzejewski 2005).

**Fig. 13:**
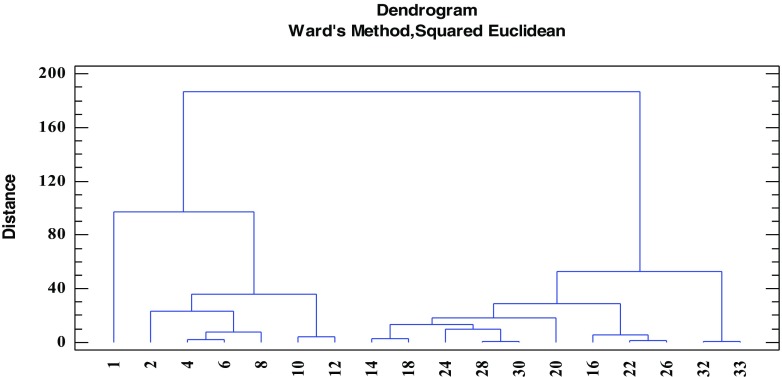
Dendrogram presenting similarity of the sediments layers in the core of the Mały Staw Lake

Considering the similarity of the studied objects (sediments’ layers in the core), two clusters are clearly distinguished, i.e., the first covering the samples deposited in years after the Second World War till present, and the second cluster containing older samples (Fig. 13). It is also evident that such division is a consequence of anthropogenic influence on the environment with the predominant influence of long-distance transport of pollutants. In the top layer of the sediments (Fig. 13 and Table 2), concentrations of the elements are substantially lower than in the adjacent layer 2. This may result from the current exchange equilibrium between the elements concentrations in the sediments and in the water body.

## Conclusions

The Mały Staw Lake is a cirque, post-glacial lake with well-documented history of natural and anthropogenic factors that influence of sediment’ properties. We proved that the comprehensive approach of the sediments’ studies, based on analytical, radiochemical, sedimentological, and geomorphological methods, confirmed environmental changes in the studied area. Based on CRS model, using ^210^Pb dating, we analyzed a 28-cm-long sediment core representing the last ca 138 years. The dating results were confirmed by ^137^Cs studies. All analytical results were presented in a time scale. For the first time, in respect to the sediments of the Mały Staw Lake, such approach was proposed and executed.

Basing on the results achieved in this study, the following could be concluded:

1. The bathymetry map has provided valuable insights into the nature and distribution of the sediments. It was also helpful in choosing the representative sampling points for the studied area;
2. Łomnica river and fans deposits deliver the solid material to the lake;
3. The causes of elevated values of sedimentation rates are a sudden huge supply materials from slopes during storm water or avalanches;
4. Taking into account the level of heavy metals concentration, the sediments were classified according to the Bojakowska scale of sediments contamination;
5. Based on historical sources and chemometric analysis, the metals’ origin were indicated and confirmed.

To summarize, the proposed research approach can be successfully applied to natural and also man-made water reservoirs studies. Particularly valuable information can be drawn with regard to man-made reservoir located in the past mining region. Furthermore, collecting information and analysis of similar records from other similar lakes in different areas of the globe could afford valuable evidence in attempt to produce more general assessment of sediments’ sensitivity to environmental changes.
